# Cellobionic acid utilization: from *Neurospora crassa* to *Saccharomyces cerevisiae*

**DOI:** 10.1186/s13068-015-0303-2

**Published:** 2015-08-16

**Authors:** Xin Li, Kulika Chomvong, Vivian Yaci Yu, Julie M Liang, Yuping Lin, Jamie H D Cate

**Affiliations:** Department of Molecular and Cell Biology, University of California, Berkeley, CA USA; Department of Plant and Microbial Biology, University of California, Berkeley, CA USA; Department of Chemistry, University of California, Berkeley, CA USA; Physical Biosciences Division, Lawrence Berkeley National Laboratory, Berkeley, CA USA

**Keywords:** Aldonic acid, LPMO, AA9, β-glucosidase, Phosphorylase, Transporter, Metabolic engineering, Biofuels, Cellobionic acid

## Abstract

**Background:**

Economical production of fuels and chemicals from plant biomass requires the efficient use of sugars derived from the plant cell wall. *Neurospora crassa*, a model lignocellulosic degrading fungus, is capable of breaking down the complex structure of the plant cell wall. In addition to cellulases and hemicellulases, *N. crassa* secretes lytic polysaccharide monooxygenases (LPMOs), which cleave cellulose by generating oxidized sugars—particularly aldonic acids. However, the strategies *N. crassa* employs to utilize these sugars are unknown.

**Results:**

We identified an aldonic acid utilization pathway in *N. crassa,* comprised of an extracellular hydrolase (NCU08755), cellobionic acid transporter (CBT-1, NCU05853) and cellobionic acid phosphorylase (CAP, NCU09425). Extracellular cellobionic acid could be imported directly by CBT-1 or cleaved to gluconic acid and glucose by a β-glucosidase (NCU08755) outside the cells. Intracellular cellobionic acid was further cleaved to glucose 1-phosphate and gluconic acid by CAP. However, it remains unclear how *N. crassa* utilizes extracellular gluconic acid. The aldonic acid pathway was successfully implemented in *Saccharomyces cerevisiae* when *N. crassa* gluconokinase was co-expressed, resulting in cellobionic acid consumption in both aerobic and anaerobic conditions.

**Conclusions:**

We successfully identified a branched aldonic acid utilization pathway in *N. crassa* and transferred its essential components into *S. cerevisiae*, a robust industrial microorganism.

**Electronic supplementary material:**

The online version of this article (doi:10.1186/s13068-015-0303-2) contains supplementary material, which is available to authorized users.

## Background

Plant biomass is a promising starting material for renewable fuel and chemical production. One of the challenges in biomass usage is the depolymerization of crystalline cellulose, a structural component of the plant cell wall. Strong inter-chain hydrogen bonds and hydrophobic interactions between cellulose sheets enable the plant cell wall to withstand harsh environmental conditions and invading microbial or animal species [1]. However, the recalcitrant nature of the plant cell wall is challenging for the enzymatic and chemical hydrolysis strategies required to release renewable sugars from plant biomass [1].

Though evolved to be resistant to degradation, the plant cell wall can be broken down by a variety of microorganisms. One important example is *Neurospora crassa,* a fungus prospering in burnt grasslands. *N. crassa* secretes cellulases and hemicellulases to degrade lignocellulosic material, thereby producing primarily shorter chain carbohydrates that can be consumed for its survival. Cellodextrin and xylodextrin utilization pathways were previously identified as major strategies used by *N. crassa* and other fungi to utilize complex biomass [2, 3]. In both cases, secreted enzymes first break down the cellulose and hemicellulose to soluble cellodextrins and xylodextrins, respectively. These are then transported into the cells by cellodextrin and xylodextrin transporters and—in the case of xylodextrins—reduced before they are further processed to monomeric sugars by intracellular hydrolases.

Recently, a new class of secreted cellulases, the copper-dependent lytic polysaccharide monooxygenases (LPMOs), classified as auxiliary activity family 9 (AA9, formerly glycosyl hydrolase family 61 enzymes GH61s), was identified [4]. LPMOs catalyze the oxidative cleavage of cellulose, generating oxidized cellodextrins, including aldonic acids, as products [5]. In their native context, they work in concert with cellobiose dehydrogenases, which provide electron equivalents to LPMOs by oxidizing cellodextrins to aldonic acids [5]. The use of LPMOs is advantageous because it enhances overall cellulose degradation and increases glucose yield [5]. Indeed, due to their ability to enhance biomass degradation, LPMOs are included in some industrial enzyme cocktails—for example, in Cellic CTec2 [6]. However, as the result of LPMO activity, the production of shorter chain aldonic acids, such as cellobionic acid and gluconic acid, is expected [6]. Although *Escherichia coli* can natively utilize gluconic acid and cellobionic acid [7, 8], the oxidized sugars cannot be utilized by *Saccharomyces cerevisiae*, a robust microorganism widely used in industrial applications. Moreover, the aldonic acids act as inhibitors of β-glucosidases in industrial cellulase cocktails, decreasing the expected glucose yield and productivity [6]. Given the widespread nature of LPMOs, it is likely that *N. crassa* consumes aldonic acids, although the pathway required to do so remains unknown.

Here, we endeavored to elucidate the aldonic acid utilization pathway in *N. crassa* and transform it into *S. cerevisiae*, to expand this yeast’s usable sugar substrates and decrease the inhibitory effects of aldonic acids on industrial cellulases.

## Results and discussion

### *Neurospora crassa* consumption of aldonic acids

As a cellulose degrading fungus, *N. crassa* is capable of utilizing Avicel, a microcrystalline cellulose. Intermediate products of Avicel utilization include cellodextrins, aldonic acids and glucose—none of which accumulated in the supernatant of *N. crassa* grown in Avicel (see Additional file 1: Figure S1). Previously, a specific cellodextrin utilization pathway was reported [2]. We hypothesized that a unique pathway responsible for aldonic acids utilization also exists in *N. crassa*.

**Fig. 1:**
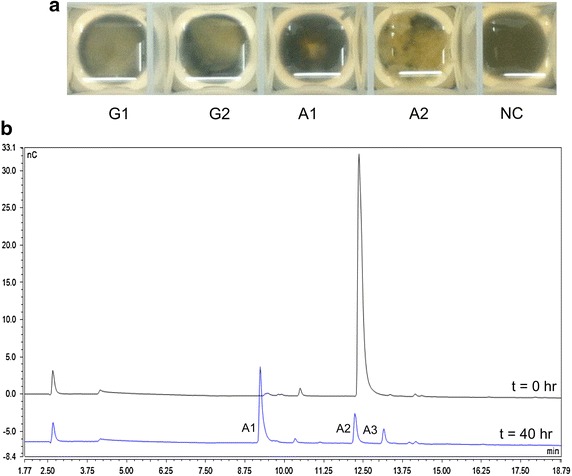
*N. crassa* growth on aldonic acids. **a** Biomass accumulation of *N. crassa* provided with different carbon sources after 48 h. All samples were started with an equal inoculum of 1 × 10^6^ cells/mL. The plate was imaged on a *black background* to highlight fungal growth. **b** Relative abundance of aldonic acids in the supernatants of cells provided with cellobionic acid at the time of inoculation (*top*) and 40 h (*bottom*). *G1* glucose, *G2* cellobiose, *A1* gluconic acid, *A2* cellobionic acid, *A3* cellotrionic acid, *NC* no carbon control.

To test for the presence of an aldonic acid utilization pathway, *N. crassa* was grown aerobically on two of the simplest aldonic acids—gluconic acid and cellobionic acid. Two days after inoculation, growth on cellobionic acid was robust while that on gluconic acid was minimal (Fig. 1a). To assess *N. crassa*’s ability to utilize aldonic acids, supernatants of the cells grown in cellobionic acid were analyzed. Comparing the starting sample to that at 40 h, the relative abundance of cellobionic acid in the media decreased (Fig. 1b). The decrease in cellobionic acid was accompanied by the appearance of gluconic acid and a small amount of cellotrionic acid, neither of which was present at the time of inoculation (Fig. 1b). These results suggested that *N. crassa* was capable of processing extracellular cellobionic acid and consuming it.

**Fig. 2:**
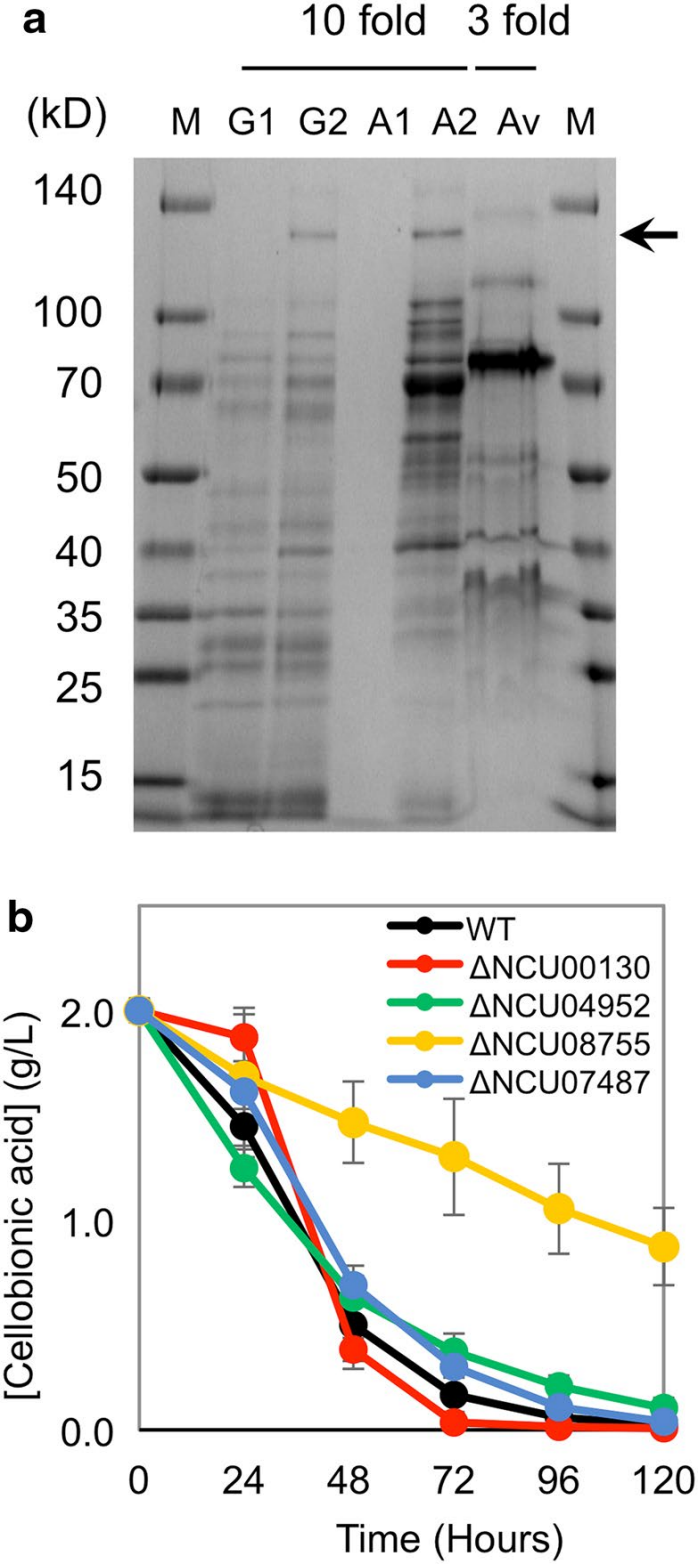
Cellobionic acid cleavage by secreted β-glucosidase NCU08755. **a** SDS-PAGE gel of proteins secreted during *N. crassa* growth on different carbon sources. The *arrow* indicates the expected size of NCU08755. *Lanes* are: molecular weight markers (M), tenfold concentrated secretomes of cultures grown on glucose (G1), cellobiose (G2), gluconic acid (A1) and cellobionic acid (A2), and a threefold concentrated secretome of a culture grown on Avicel cellulose (Av). **b** Cellobionic acid consumption profile of the four *N. crassa* strains with secreted β-glucosidases individually deleted.

We next tested whether the β-1,4 glycosidic bond in cellobionic acid is targeted by β-glucosidase family enzymes. The *N. crassa* genome encodes at least seven β-glucosidases, four of which are highly upregulated when *N. crassa* is grown on cellulose [9]. To identify β-glucosidases responsible for degrading cellobionic acid, the secretome of *N. crassa* grown on cellobionic acid was analyzed by LC–MS/MS. Only one of the four major β-glucosidases, NCU08755, was identified in the secretome of cells grown in cellobionic acid (Fig. 2a, see Additional file 1: Figure S2). The protein band for NCU08755 was absent in the secretome of cells grown on gluconic acid (Fig. 2a). We then tested cellobionic acid consumption by strains of *N. crassa* with the four β-glucosidases knocked out individually. Only the *ΔNCU08755* strain showed a decrease in cellobionic acid consumption in comparison to the wild-type strain (Fig. 2b). These results suggested that NCU08755 is the major β-glucosidase involved in cellobionic acid depolymerization in *N. crassa*. In addition, a phylogenetic analysis of NCU08755 fungal homologs suggested that extracellular cellobionic acid cleavage might be a common strategy among fungi (see Additional file 1: Figure S3).

### Identification of the cellobionic acid transporter in *N. crassa*

**Fig. 3:**
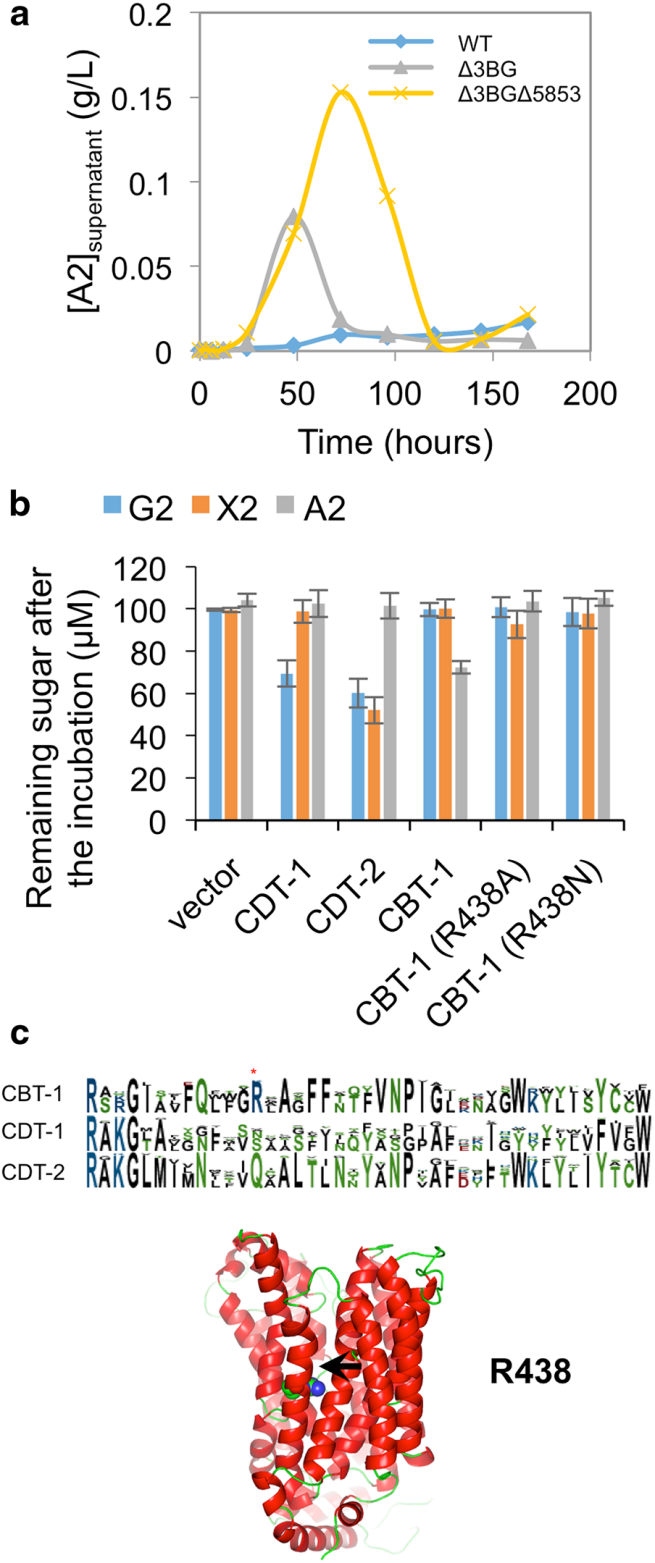
Specific cellobionic acid transporter NCU05853 (CBT-1). **a** Accumulation of cellobionic acid (A2) in the supernatant of β-glucosidase and NCU05853 mutant strains, providing Avicel as carbon source. Δ3BG, triple β-glucosidase deletion strain; Δ3BGΔ5853, triple β-glucosidase deletion plus *ΔNCU05853* deletion strain. **b** Percentage of the remaining sugars after incubation with *S. cerevisiae* strains expressing different transporters. **c** Homology model of CBT-1, showing the conserved arginine residue R438. *G2* cellobiose, *X2* xylobiose, *A2* cellobionic acid.

Deletion of NCU08755 slowed *N. crassa* growth on cellobionic acid but did not eliminate it, which implies that *N. crassa* expresses a parallel intracellular depolymerization pathway. Indeed, in a recent analysis of the *N. crassa* phosphoproteome, we identified a cellobionic acid transporter (NCU05853, or CBT-1) [10], a major facilitator superfamily (MFS) transporter previously noted to be highly upregulated in cellulolytic conditions [11], and recently identified as important in cellulase induction [12]. We investigated whether NCU05853 is the predominant cellobionic acid transporter in *N. crassa* using the triple extracellular β-glucosidase knockout background *Δ3BG* (*ΔNCU08755*, *ΔNCU00130* and *ΔNCU04952*). In the *Δ3BG* strain, providing Avicel as carbon source, cellobionic acid accumulated as expected because this strain lacks the extracellular β-glucosidase NCU08755 responsible for cellobionic acid cleavage to gluconic acid and glucose (Fig. 3a). Notably, when *NCU05853* was also deleted in addition to the three extracellular β-glucosidases, extracellular cellobionic acid accumulated in the growth media to a much higher level (Fig. 3a). To investigate the substrate specificities of NCU05853, the transporter was cloned and expressed in *S. cerevisiae.* Transport assays showed that only NCU05853, but not the previously studied NCU00801 (CDT-1) nor NCU08114 (CDT-2), was capable of transporting cellobionic acid into the cell (Fig. 3b). However, NCU05853 was not capable of transporting cellobiose or xylobiose (Fig. 3b). These results suggest that NCU05853 (CBT-1, hereafter) is a specific cellobionic acid transporter.

Multiple sequence alignments of CDT-1, CDT-2 and CBT-1 homologs revealed a conserved arginine residue R438 specific to CBTs, which is located near the substrate-binding pocket of the CBT-1 homology model (Fig. 3c). Replacement of R438 with either alanine or asparagine to remove the positive charge (R438A and R438N) eliminated the ability of CBT-1 to transport cellobionic acid completely (Fig. 3b). These results identified R438 as a key residue responsible for CBT-1 substrate specificity.

In addition to CBT-1, given the function of the secreted NCU08755, *N. crassa* may also have a transporter or a permease responsible for gluconic acid import. However, due to the fact that *N. crassa* grew poorly on gluconic acid, we did not investigate further to identify gluconic acid transporters.

### Identification of the cellobionic acid phosphorylase

**Fig. 4:**
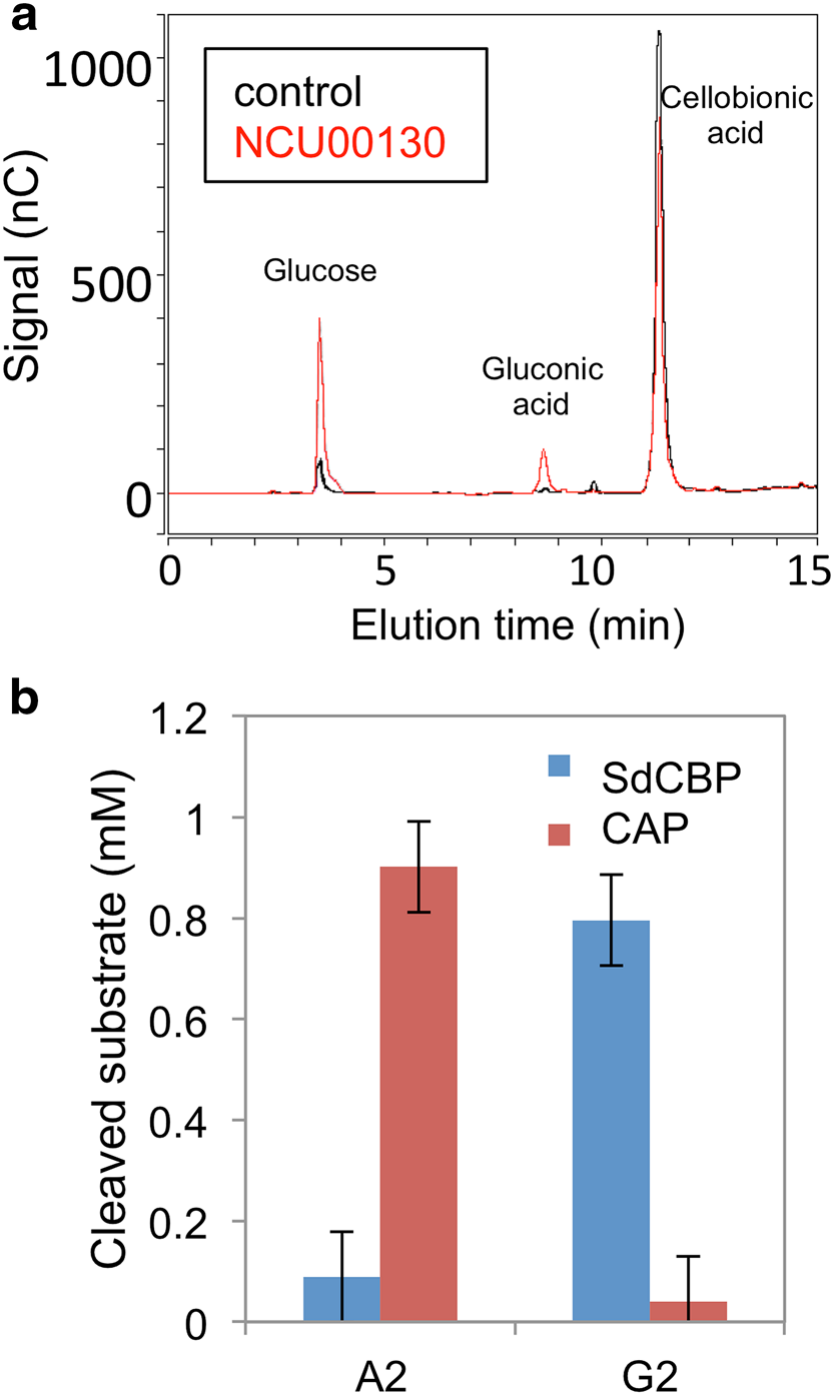
Cellobionic acid phosphorylase NCU09425 (CAP). **a** In vitro activity assay of purified intracellular β-glucosidase *gh1*-*1* (NCU00130) on cellobionic acid. The purified NCU00130 was omitted in the ‘control’ condition. **b** In vitro activity assay of purified NCU09425 (CAP) and *Saccharophagus degradans* cellobiose phosphorylase (SdCBP) on cellobionic acid and cellobiose. *A2* cellobionic acid, *G2* cellobiose.

After cellobionic acid is transported into *N. crassa*, it must be processed to monomeric units for further utilization. An intracellular β-glucosidase NCU00130 was previously reported to cleave cellobiose to two molecules of glucose [2]. Purified NCU00130 was tested for its ability to hydrolyze cellobionic acid, and was able to release gluconic acid from cellobionic acid (Fig. 4a). However, the reaction rate was poor (with apparent turnover number of 0.11 s^−1^) in comparison to reactions with cellobiose as the substrate [13]. We hypothesized that *N. crassa* likely uses another pathway to consume cellobionic acid rather than relying on NCU00130 activity.

Although *N. crassa* utilizes cellodextrin via a hydrolytic pathway, bacteria employ a different intracellular enzyme, namely a phosphorylase, to cleave cellobiose to glucose and glucose 1-phosphate [14]. Cellobiose phosphorylase homologs can be found in multiple cellulosic fungi, including in *N. crassa* with protein NCU09425. Prior work identified NCU09425 as a cellobionic acid phosphorylase [15]. When NCU09425 was co-expressed with CDT-1 in *S. cerevisiae*, cellobiose consumption was not observed (see Additional file 1: Figure S4), indicating that NCU09425 may be specific for cellobionic acid. Consistent with this idea, NCU09425 expressed in and purified from *S. cerevisiae* specifically cleaved cellobionic acid to gluconic acid and glucose 1-phosphate (Fig. 4b) [15], but had no activity on cellobiose (Fig. 4b). These results indicate that NCU09425 (CAP, hereafter) is a specific cellobionic acid phosphorylase, responsible for intracellular cellobionic cleavage for further consumption. This specificity is consistent with a recent crystal structure and mutational analysis of a bacterial homolog to CAP [16], and the lack of cellobionic acid utilization in *ΔNCU9425 N. crassa* [17].

### Engineering the cellobionic acid utilization pathway in *S. cerevisiae*

**Fig. 5:**
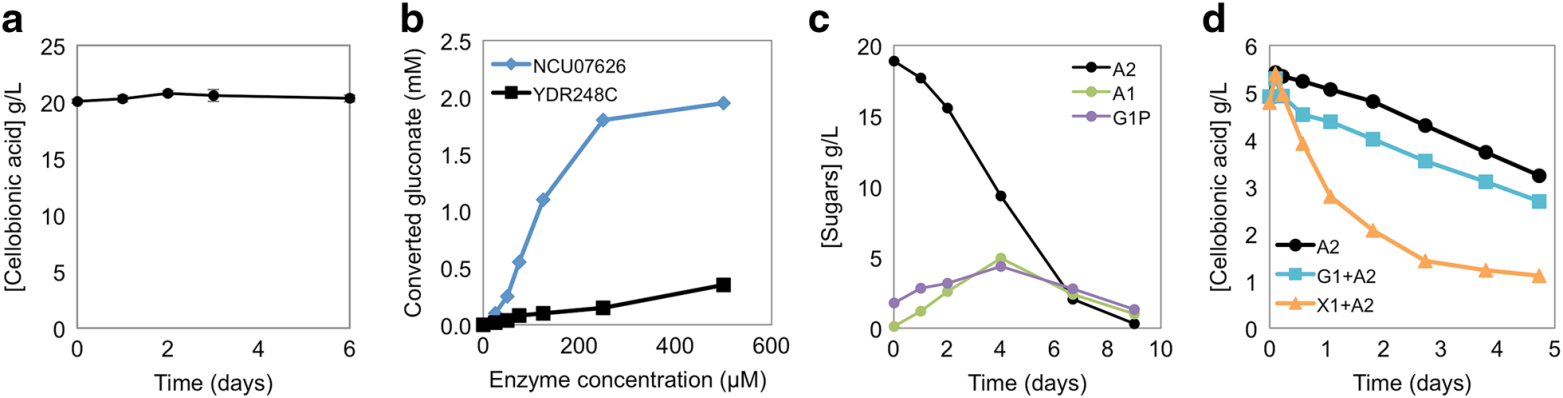
Reconstituted cellobionic acid utilization pathway in *S. cerevisiae.* **a** Aerobic cellobionic acid consumption of *S. cerevisiae* expressing CBT-1 and CAP. **b** In vitro assays of the purified gluconokinases from *N. crassa* (NCU07626, GnK) and *S. cerevisiae* (YDR248C). **c** Aerobic cellobionic acid consumption of *S. cerevisiae* expressing CBT-1, CAP and GnK. **d** Anaerobic cellobionic acid consumption of *S. cerevisiae* expressing CBT-1, CAP and GnK, providing cellobionic acid and glucose or xylose as an additional carbon source.

With the identification and characterization of the cellobionic acid transporter (CBT-1) and the intracellular cellobionic acid phosphorylase (CAP), we aimed to transfer the cellobionic acid pathway from *N. crassa* to the industrial yeast *S. cerevisiae*. However, *S. cerevisiae* expressing CBT-1 and CAP did not consume cellobionic acid (Fig. 5a). Since the activity assays of the CBT-1 and CAP revealed that they were both functionally expressed in *S. cerevisiae*, the failure to consume cellobionic acid indicated that there are likely other components in the cellobionic acid utilization pathway missing in *S. cerevisiae*. Whereas the glucose 1-phosphate released by CAP is likely consumed directly by conversion to glucose 6-phosphate by phosphoglucomutase (Pgm2 or Pgm1 in yeast), we hypothesized that the activity of the putative endogenous *S. cerevisiae* gluconokinase (YDR248C) responsible for converting gluconic acid to 6-phosphogluconate was limited, resulting in the failure of the cellobionic acid consumption pathway to function. To test this hypothesis, gluconokinases from *S. cerevisiae* (YDR248C) and *N. crassa* (NCU07626) were purified and tested for activity in vitro. In comparison to YDR248C, *N. crassa* gluconokinase (GnK, hereafter) was capable of converting more gluconic acid to 6-phosphogluconate at all enzyme concentrations tested (Fig. 5b). When GnK was co-expressed along with CBT-1 and CAP in *S. cerevisiae*, cellobionic acid consumption was observed in aerobic conditions (Fig. 5c). These results suggest that gluconokinase activity in *S. cerevisiae* was limiting the cellobionic acid pathway. Notably, even though YDR248C has gluconokinase activity in vitro and is annotated to be a cytoplasmic protein [18], its pattern of co-expression suggests that it is more likely to be a mitochondrially targeted protein (SGD co-expression analysis, [19]).

**Fig. 6:**
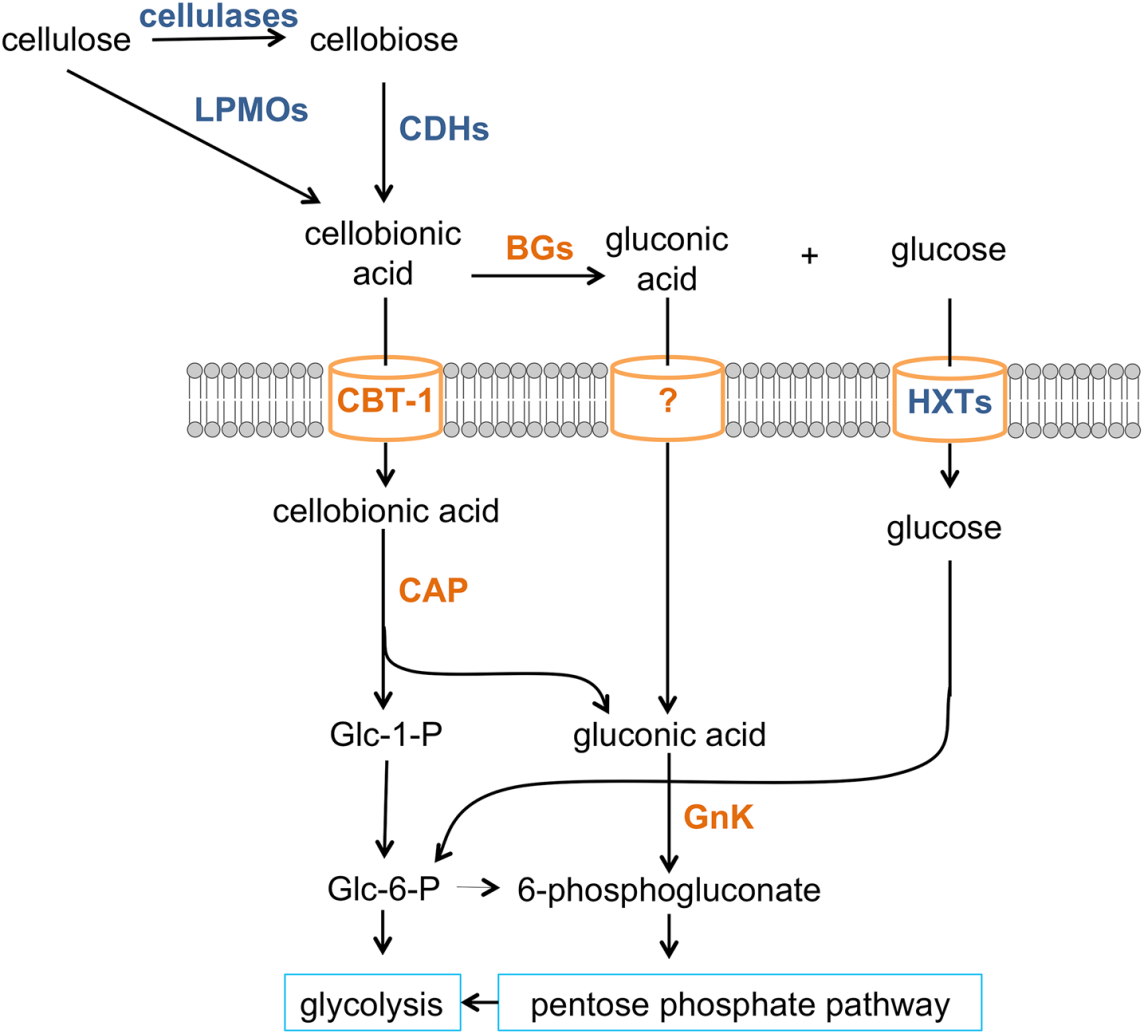
Aldonic acid consumption pathway. Schematic of aldonic acid utilization in *N. crassa* and *S. cerevisiae*. Enzymes are abbreviated as follows: *LPMOs* lytic polysaccharide monooxygenases, *CDHs* cellobiose dehydrogenases, *BGs* β-glucosidases, *CBT-1* cellobionic transporter, *HXTs* hexose transporters, *CAP* cellobionic acid phosphorylase, *GnK* gluconokinase.

Although *S. cerevisiae* was capable of utilizing cellobionic acid in aerobic conditions, its consumption in anaerobic conditions was poor. We hypothesized that co-utilization of cellobionic acid with other sugars might improve the anaerobic consumption rates, similar to the phenomenon observed in cellobiose–xylose and xylodextrin–xylose co-consumption experiments [3, 20, 21]. To test this hypothesis, either glucose or xylose was provided in addition to cellobionic acid for anaerobic fermentations of the engineered yeast strains expressing CBT-1, CAP and GnK. The rate of cellobionic acid consumption improved ~5.5-fold in the presence of xylose (Fig. 5d). Although the rate of cellobionic acid consumption remained unchanged when glucose was provided, the lag phase of cellobionic consumption was shortened by ~12 h (Fig. 5d). These results suggest that the addition of other sugars, particularly xylose, can have positive effects on anaerobic consumption of cellobionic acid in *S. cerevisiae*. This may be because cellobionic acid consumption was previously limited by the rate of gluconic acid utilization via 6-phosphogluconate and the pentose phosphate pathway (Fig. 6). Addition of xylose may improve flux through the pentose phosphate pathway, increasing the ability of *S. cerevisiae* to utilize gluconic acid.

## Conclusions

In this study, we successfully identified a novel aldonic acid utilization pathway in *N. crassa* (Fig. 6). Cellobionic acid that would be released from cellulose via LPMOs and by CDHs was hydrolyzed by the secreted NCU08755 β-glucosidase to glucose and gluconic acid. This activity was specific to NCU08755 among the four major secreted β-glucosidases. Deletion of NCU08755 decreased but did not eliminate cellobionic acid consumption, suggesting that *N. crassa* was capable of consuming both cellobionic acid and gluconic acid and that transporters for each must exist. The cellobionic acid transporter (NCU05853, CBT-1) was identified in this study. However, we were not successful in identifying the gluconic acid transporter. For intracellular cellobionic acid consumption, cellobionic acid was cleaved to glucose 1-phosphate and gluconic acid by the cellobionic acid phosphorylase (NCU09425, CAP). These products would then be available to glycolysis and the pentose phosphate pathway, respectively. Glucose 1-phosphate would be converted to glucose 6-phosphate, whereas gluconic acid required conversion to 6-phosphogluconate by gluconokinase (GnK) to be utilized by the pentose phosphate pathway prior to entering glycolysis.

Our discovery of the native *N. crassa* aldonic acid consumption pathway and the successful transfer of the pathway from *N. crassa* to *S. cerevisiae* should prove beneficial to economical biomass utilization. Although aldonic acids can be utilized by *E.coli* as previously shown [7, 8], this study is the first, to the best of our knowledge, to demonstrate cellobionic acid utilization in the widely used industrial host, *S. cerevisiae*. *S. cerevisiae* is suitable for large-scale biofuel production because it circumvents phage and other bacterial contamination issues encountered in *E. coli*-based production. The cellobionic acid utilization pathway described here is the third plant cell wall consumption pathway from *N. crassa* reconstituted in *S. cerevisiae*, in addition to the cellodextrin and the xylodextrin utilization pathways [2, 3]. By applying all the three pathways simultaneously, *S. cerevisiae* could be turned into a ‘voracious’ yeast, capable of consuming substantial fractions of the plant cell wall in biomass hydrolysates.

## Methods

### Cellobionic acid preparation

Cellobionic acid was synthesized by a modified Frush and Isbell procedure [22]. In 500 mL ice-cold water, 0.075 mol of cellobiose was added with 0.15 mol CaCO_3_ and 0.090 mol bromine. The reaction was stirred at room temperature in the dark for 4 h, after which residual bromine was removed by purging the solution with nitrogen gas for 1 h. The product was then concentrated on a rotary evaporator at 50 °C to about 50 % wt/vol concentration. Purification was performed on a home-packed Supelclean™ ENVI-Carb™ SPE (Sigma-Aldrich) column with an elution gradient from water to 50 % acetonitrile. Purity was confirmed by high-performance liquid chromatography (HPLC).

### *N. crassa* strains

*Neurospora crassa* strains obtained from the Fungal Genetics Stock Center (FGSC) [23] include the WT (FGSC 2489), and deletion strains for the three oligosaccharide transporters: NCU00801 (FGSC 16575, *Δcdt*-*1*), NCU08114 (FGSC 17868, *Δcdt*-*2*), and NCU05853 (FGSC 13770, *ΔNCU05853*). The Δ3BG strain lacking all three major β-glucosidase (NCU00130, NCU04952, and NCU08755) genes, and the Δ3BGΔCDT1, Δ3BGΔCDT2 and Δ3BGΔ5853 strains were described previously [9].

### *N. crassa* growth assay

Conidia were inoculated at a concentration equal to 10^6^ conidia per milliliter in 3 mL Vogel’s salts [24] with 1 % wt/vol Avicel PH 101 (Sigma), glucose (Sigma), cellobiose (Sigma), gluconic acid (Sigma), cellobionic acid prepared as described above, or with no carbon, in a 24-well deep-well plate. The media was neutralized to pH = 5.8. The plate was sealed with Corning™ breathable sealing tape and incubated at 25 °C in constant light and shaking (200 rpm). Images were taken at 48 h after inoculation. Culture supernatants were diluted 200 times with 0.1 M NaOH before Dionex HPLC analysis.

### Heterologous expression plasmids and yeast strains

Template gDNA from *N. crassa* WT strain (FGSC 2489) and from *S.cerevisiae* S288C strain was extracted according to the method of Lee and Taylor (http://www.fgsc.net/fgn35/lee35.pdf) and Harju et al. [25], respectively. Open reading frames (ORFs) of the cellobionate phosphorylase (NCU09425), and gluconate kinase (NCU07626) genes were amplified from *N. crassa* template. The yeast gluconate kinase (YDR248C) ORF was amplified from an *S. cerevisiae* gDNA template. Each ORF fused with C-terminal His_6_-tag was flanked with the *S. cerevisiae* TEF1 promoter and CYC transcriptional terminator in the 2µ yeast plasmid pRS423 backbone. The plasmids pRS425_NCU00130, and pRS426_NCU05853 were described previously [2]. Plasmid pAA3, a single-plasmid form of the cellobionic acid pathway, was constructed by integrating NCU05853, NCU09425, and NCU07626 into the pRS425 backbone. Plasmid pAA10 was constructed by replacing the promoter of each of NCU05853, NCU09425, and NCU07626 to P_*CCW12*_. *S. cerevisiae* strain D452-2 (*MAT***a***leu2 his3 ura3 can1*) [26] was used as the recipient strain for the cellobionic acid only fermentation. Strain SR8 [27], an evolved xylose utilization *S. cerevisiae* strain, was used as the recipient strain for all the co-fermentation experiments. The plasmid encoding the CDT-1 (F213L) mutant with improved maximal cellobiose transport rate [13] was co-transformed with plasmids encoding cellobiose phosphorylase from *S. degradans* [28] or NCU09425 for aerobic growth assays on cellobiose.

### Yeast cell-based cellobionic acid uptake assay

*Saccharomyces cerevisiae* was grown in an optimized minimum medium (oMM) [29] lacking uracil into late log phase. Cells were then harvested and washed three times with the assay buffer (5 mM MES, 100 mM NaCl, pH 6.0) and resuspended to final OD of 40. Substrate stocks were prepared in the same assay buffer with a concentration of 200 μM cellobiose, xylobiose, or cellobionic acid. Transport assays were initiated by mixing equal volumes of cell suspension and substrate stock. Reactions were incubated at 30 °C with continuous shaking for 30 min. Samples were centrifuged at 14,000 rpm at 4 °C for 5 min. 400 μL of each sample was transferred to an HPLC vial containing 100 μL 0.5 M NaOH. The concentration of remaining cellobionic acid or cellobiose was measured by high-performance anion exchange chromatography (HPAEC).

### Enzyme purification

*Saccharomyces cerevisiae* strains transformed with enzyme-expression plasmids (pRS423_NCU09425, pRS423_NCU07626, or pRS423_YDR248C) were grown in oMM media lacking histidine until late log phase before harvesting by centrifugation. The yeast cell pellet was resuspended in a buffer containing 50 mM Tris–HCl, 100 mM NaCl, 0.5 mM DTT, pH 7.4 and protease inhibitor cocktail (Pierce). Cells were lysed with an Avestin homogenizer (Avestin, Inc.). Clarified supernatant was load onto a HisTrap column (GE Healthcare), and the His_6_-tagged enzymes were eluted with an imidazole gradient. Purified enzymes were buffer exchanged to 20 mM Tris–HCl, 100 mM NaCl, pH 7.4, and concentrated to 5 mg/mL. NCU00130 from *N. crassa* [2] and cellobiose phosphorylase from *Saccharophagus degradans* [28] were expressed and purified as previously described.

### Enzyme assays

For the β-glucosidase activity assay, 0.5 μM purified NCU00130 was incubated with 500 μM cellobionic acid in 1x PBS at 30 °C for 30 min. Reactions were quenched by adding 10 volumes of 0.1 M NaOH and analyzed with HPAEC. For the cellobionate phosphorylase activity assay, 0.5 μM NCU09425 was incubated with 2 mM cellobionate or cellobiose in 1x PBS at 30 °C for 30 min. Reactions were quenched by adding 10 volumes of 0.1 M NaOH and analyzed with HPAEC. For the gluconate kinase assay, 0.2 μM NCU07626 or YDR248C was incubated with 1 mM gluconate and 5 mM ATP in 1x PBS at 30 °C for 30 min and the products were analyzed by HPAEC.

### HPAEC analysis

HPAEC analysis was performed on a ICS-3000 HPLC (Dionex Corporation) using a CarboPac PA200 analytical column (3 × 150 mm) and a CarboPac PA200 guard column (3 × 30 mm) at 30 °C. Following injection of 25 μL of diluted samples, elution was performed at 0.4 mL∕ min using 0.1 M NaOH in the mobile phase with sodium acetate gradients. For aldonic acid separation, the acetate gradients were 0 mM for 1 min, increasing to 80 mM in 8 min, increasing to 300 mM in 2 min, keeping at 300 mM for 1 min, increasing to 600 mM for 2 min, keeping at 600 mM for 5 min, and re-equilibrating at 0 mM for 4 min. Elution was monitored using pulsed amperometric detection (PAD) and peaks were analyzed and quantified using the Chromeleon software package.

### Sequence alignment and homology modeling

Homologs of CBT-1, CDT-1, and CDT-2 were found with BLAST [30] queries against NCBI non-redundant protein sequence database with E value cutoff at 1E−130. Multiple sequence alignments were carried out using T-COFFEE software package with default parameters [31]. Sequence logos were created with WebLogo [32].

Homology models of CBT-1 were built with Modeller v9.12 [33] using a xylose transporter XylE structure; PDB code 4GBZ [34] as the template. Figure 3c was prepared with the molecular visualization software PyMol (http://www.pymol.org).

## Additional file

Additional file 1. Supplementary figures.

## Abbreviations

LPMO: lytic polysaccharide monooxygenase
GH61: glycosyl hydrolase family 61
BG: β-glucosidase
CDT: cellodextrin transporter
CBT-1: cellobionic acid transporter
CAP: cellobionic acid phosphorylase
GnK: gluconokinase
