# Supplementary material for: Cellobionic acid utilization: from Neurospora crassa to Saccharomyces cerevisiae
Source: Biotechnol Biofuels. 2015 Aug 16;8:120. doi: 10.1186/s13068-015-0303-2 (PMC4537572; doi:10.1186/s13068-015-0303-2)
Supplement: Additional file 1. — Supplementary figures. [file 13068_2015_303_MOESM1_ESM.pdf]

**Additional File 1**

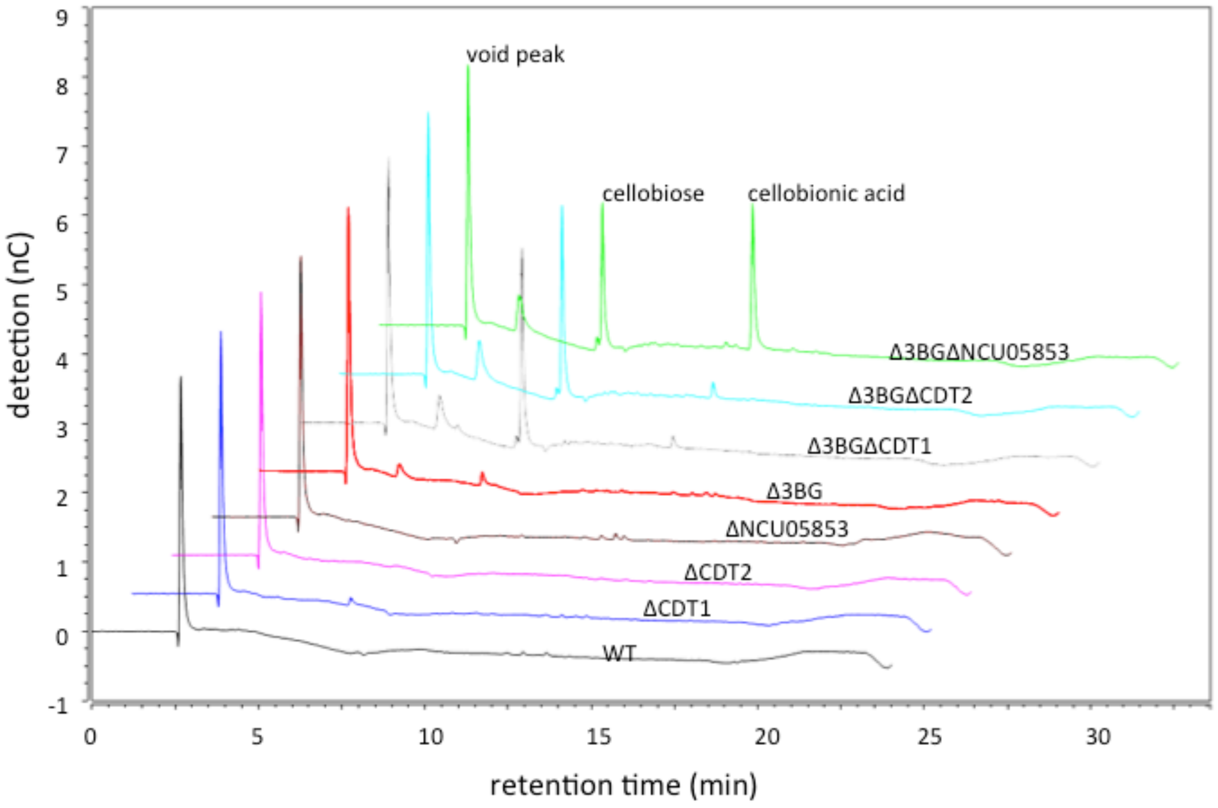


**Figure S1. Carbohydrate analysis of 5-day Avicel medium supernatant.** Relative abundance of sugar species in the supernatant of various *N. crassa* wild-type and mutant strains grown on Avicel. Strains are indicated in the Materials and Methods.

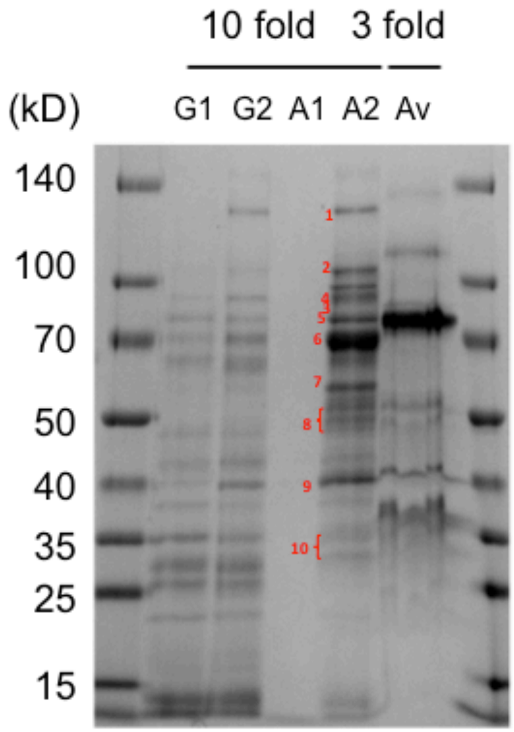


| | | |
|---|---|---|
| 1 | NCU08755 | beta-glucosidase 1 |
| 2 | NCU07523 | 1,3-beta glucanase |
| 3 | NCU06512 | methionine synthase |
| 4 | NCU04265 | hypothetical protein |
| | NCU00798 | hypothetical protein |
| | NCU00355 | catalase-3 |
| 5 | NCU05137 | non-anchored cell wall protein-1 |
| 6 | NCU01517 | glucoamylase precursor |
| 7 | NCU04674 | alpha-glucosidase |
| | NCU09175 | GPI-anchored cell wall beta-1,3-endoglucanase EglC |
| | NCU08936 | clock-controlled gene-15 |
| 8 | NCU08746 | starch binding domain-containing protein |
| | NCU09659 | 5'-nucleotidase |
| | NCU01395 | WSC domain-containing protein |
| 9 | NCU08171 | anchored cell wall protein-12 |
| | NCU07190 | endoglucanase 3 |
| | NCU08760 | GH61, PMO-1 with CBM, upregulated by Avicel |
| 10 | NCU05974 | cell wall glucanosyltransferase Mwg1 |
| | NCU05789 | secreted glucosidase |
| | NCU02136 | transaldolase |
| | NCU04431 | endo-1,3(4)-beta-glucanase |

**Figure S2. Identification of secreted proteins of *N. crassa* cultures grown on cellobionic acid.** SDS-PAGE gel of the secretome (L) was analyzed by MS/MS (R). Gene names and protein annotation are given for the numbered bands.

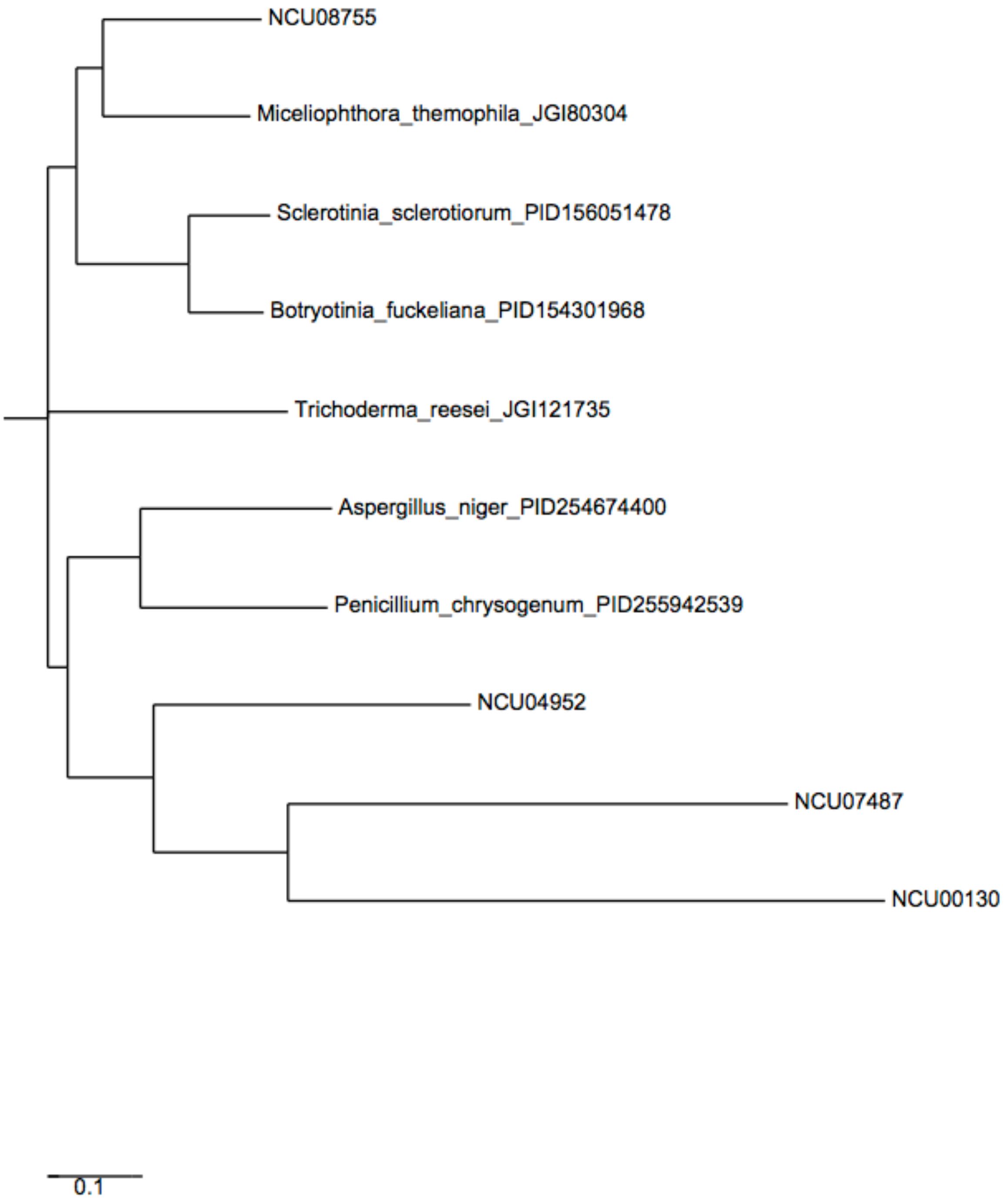


**Figure S3 Phylogenetic analysis of β-glucosidase NCU08755.** Homologous proteins from other fungal species are shown, along with the other three secreted β-glucosidases from *N. crassa*.

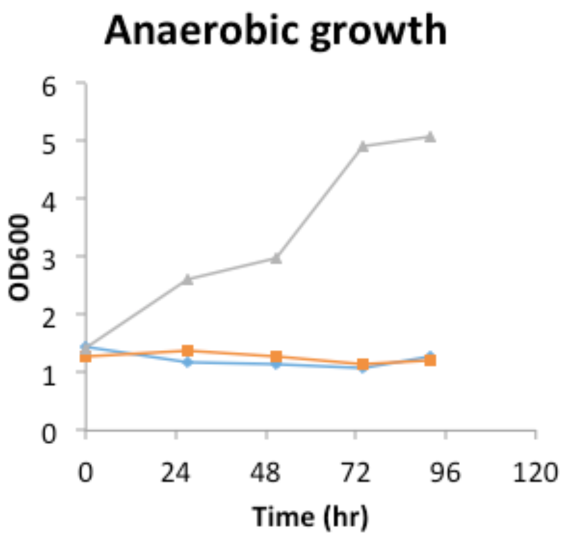


**Figure S4. Putative *N. crassa* cellobionic acid phosphorylase (CAP, NCU09425) lack of activity on cellobiose.** (A) Optical density of *S. cerevisiae* cultures expressing CDT-1 (F213L) and cellobiose phosphorylase (CBP, grey) or NCU09425, grown on cellobiose. (B) Cellobiose concentrations remaining in the culture broth of the cultures in (A). Plasmids in the various strains are as follows: 425+426, pRS425 and pRS426 empty vectors as controls; cdt1F213L NCU09425, plasmids expressing transporter CDT-1(F213L) and *N. crassa* protein NCU09425; cdt1F213L SdCBPWT, plasmids expressing transporter CDT-1(F213L) and *S. degradans* cellobiose phosphorylase (CBP).
